# Trueness of intraoral scanners according to subgingival depth of abutment for fixed prosthesis

**DOI:** 10.1038/s41598-022-23498-x

**Published:** 2022-12-01

**Authors:** Young-Tak Son, KeunBaDa Son, Kyu-Bok Lee

**Affiliations:** 1 Department of Dental Science, Graduate School, Kyungpook National University, Daegu, Republic of Korea; 2 Advanced Dental Device Development Institute, Kyungpook National University, Daegu, Republic of Korea; 3 Department of Prosthodontics, School of Dentistry, Advanced Dental Device Development Institute, Kyungpook National University, 2177 Dalgubuldaero, Jung-Gu, Daegu, 41940 Republic of Korea

**Keywords:** Health care, Medical research

## Abstract

This study aimed to compare the trueness of intraoral scanners (IOSs) according to the subgingival finish line depth of tooth preparation for fixed prostheses. The prepared maxillary right first molar was fabricated by using ceramic material. A computer-aided design (CAD) reference model (CRM) of the abutment was obtained by using a contact scanner. The subgingival finish line was located according to the depth at 0-mm, 0.25-mm, 0.5-mm, 0.75-mm, and 1-mm. CAD test models (CTMs) were obtained by using 2 IOSs (i500 and CS3600). CRM and CTM were superimposed and analyzed (Geomagic control X). The one-way analysis of variance (ANOVA) was used to compare the trueness according to the subgingival finish line depth. The paired t test was used to compare the trueness of IOSs with and without gingival retraction (α = .05). When the gingival displacement code was not used, it was observed that the trueness of both IOSs decreased significantly as the depth of the subgingival finish line increased (*P* < 0.001). When the subgingival finish line was positioned deeper than 0.5-mm, the trueness of both IOSs exceeded 100 µm in the marginal region. When the gingival displacement cord was used, the trueness of both IOSs did not exceed 100 µm regardless of the subgingival finish line depth. When gingival cord was used, it showed significantly higher trueness than when not used (*P* < 0.001). When the gingival displacement cord was not used, the trueness of IOSs decreased as the subgingival finish line depth increased. But the use of the gingival displacement cord improved the scanning trueness by 90%. Thus, it is necessary to use the gingival displacement cord according to the clinical situation to improve scan trueness at the subgingival finish line.

## Introduction

The advent of intraoral scanners (IOSs), computer-aided design, and computer-aided manufacturing (CAD/CAM) has enabled complete digital workflow^1,2^. Accurate digital scans in digital workflow are an important factor for the success of prostheses^3–7^. However, if accurate scans are not performed, it may take a lot of time to adjust the prosthesis, and it may be necessary to refabricate the prosthesis in severe cases^8,9^.

The accuracy of IOSs may vary depending on various conditions, including accuracy of the scanning method^10,11^, effect of lighting conditions on the scanning accuracy^12,13^, accuracy depending on the IOS type^3,4,14^, distortion that occurs when using IOSs^15,16^, and interference of saliva^17,18^. Additionally, in another study, the difference in scan accuracy was evaluated according to the finish line position of the tooth preparation abutment^19–21^. Based on the location of the finish line, the scan data revealed that the supragingival finish line showed better accuracy and the subgingival finish line showed inaccurate accuracy^19,21^. These results indicate that the location of the finish line can affect the accuracy of the IOS. However, studies on the accuracy of IOSs according to the depth of various subgingival finish lines are still insufficient.

In current clinical situations, various locations of the finish line are required for tooth preparation for fixed prostheses based on the purpose of treatment^22–27^. Various depths of subgingival finish lines can be applied according to the patient treatment plan during tooth preparation for a fixed prosthesis in clinical situations^28–30^. However, in previous studies, it has been shown that normal extension to the gingival sulcus should not exceed 0.5-mm to 1-mm^22,23,29,30^. Positioning the finish line at a depth greater than 1-mm is a serious error and can cause epithelial attachment tissue damage and gingivitis^29,30^.

The impression of the inaccurate finish line negatively affects the fit of the prosthesis, and periodontitis, dental caries, and fracture of the prosthesis may occur^31,32^. Previous studies have reported that the accuracy of IOS exceeding 100 μm negatively affects the marginal and internal fit of fixed prostheses^6,33^. Another study has reported that the scan accuracy is recommended to be less than 100 μm considering the cement space of the fixed prosthesis^34^. Additionally, in the case of subgingival finish lines, gingival retraction was recommended for accurate impressions^1,9,19–21,33^. The gingival displacement cords dislocate the gingival margin apically and expand the gingival sulcus to increase the surface of the abutment that the IOS light beam can reach.^9^ When gingival retraction was used, the accuracy of the impression was found to be increase^19,21^. This indicates that the presence or absence of gingival retraction can affect the accuracy of impression.

Therefore, this study aimed to compare the trueness of IOSs according to the finish line depth (0-mm, 0.25-mm, 0.5-mm, 0.75-mm, and 1-mm) of tooth preparation for fixed prostheses and to compare the trueness of IOSs with and without gingival retraction. The first null hypothesis is that the trueness of IOSs does not change based on the subgingival finish line depth of tooth preparation. The second null hypothesis is that the trueness of IOSs does not change with and without gingival retraction.

## Methods

To determine the number of samples per group, 5 pilot experiments were performed prior to the present study, and 20 per group were determined based on the following results using a power analysis software (G*Power v3.1.9.2; Heinrich-Heine-Universität, Düsseldorf, Germany): effect size (f) = 0.52; power = 99%; actual power = 99.20%.

**Figure 1:**
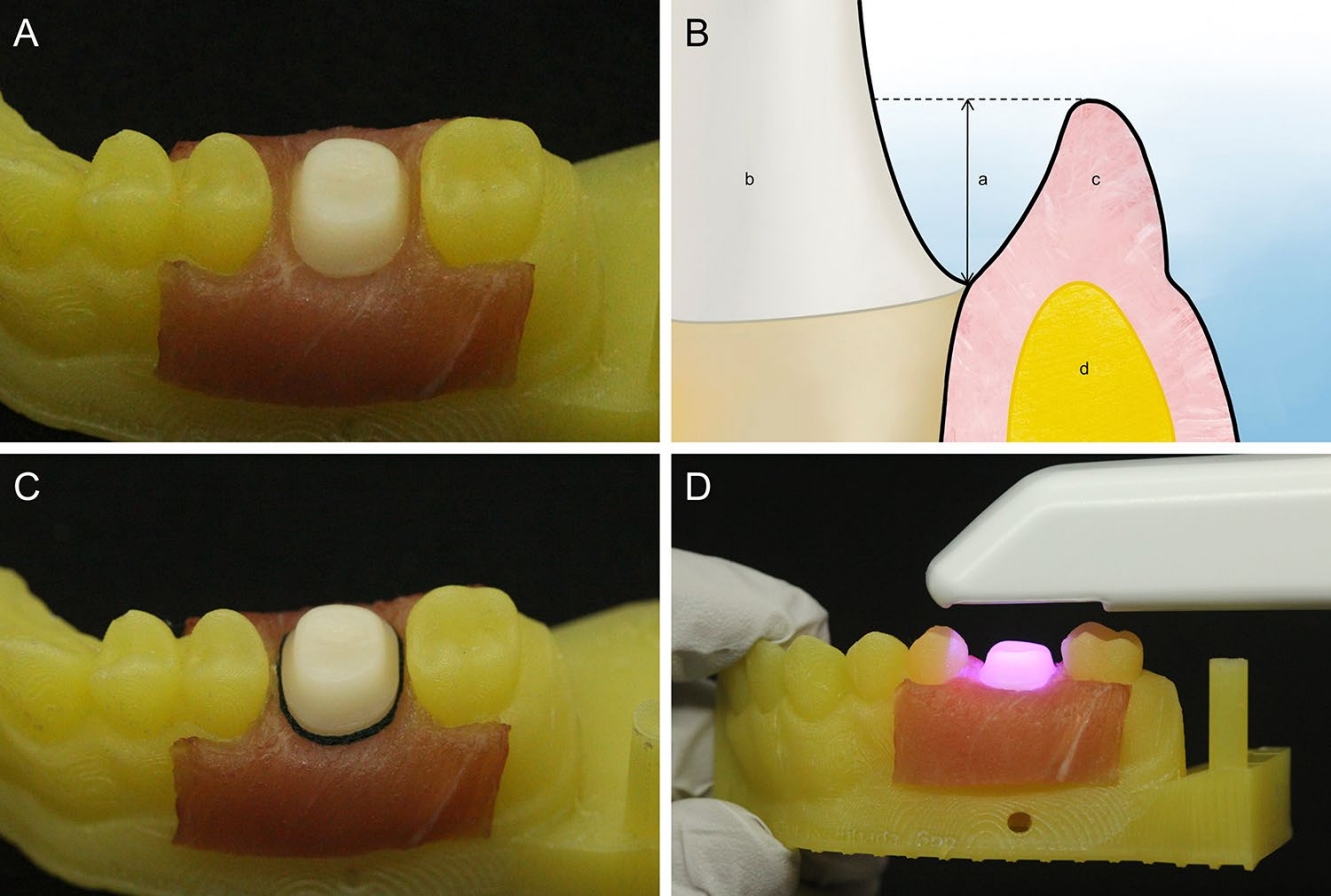
Reference model and scanning process. (**A**) Model with artificial gingiva formed. (**B**) Schematic of subgingival finish line. (a) Depth of finish line. (b) Abutment. (c) Artificial gingiva. (d) 3D printed model. (**C**) Gingival displacement cord inserted in artificial gingiva. (**D**) Intraoral scanning for CTM fabrication.

The maxillary right first molar of the typodont model (PRO2001-UL-SP-FEM-32; Nissin Dental Product, Kyoto, Japan) was used for the fabrication of the reference model. Tooth preparation was performed under the following conditions: reduction of 1.5 mm in the occlusal direction, reduction of 1.2 mm in the axial direction with chamfer shape of finish line. The tooth preparation model was scanned by using a laboratory scanner (E1; 3Shape, Copenhagen, Denmark). The scanned model was formed in standard tessellation language (STL). The formed STL file was saved separately for the abutment and adjacent teeth. A 3D printer (Meg-printer 2; Megagen, Daegu, Republic of Korea) was used to fabricate the adjacent teeth, except the abutment. The abutment was fabricated as a reference model by using the milling equipment (EZIS HM; DDS, Seoul, Republic of Korea). For abutment teeth, lithium disilicate ceramic (IPS e.max CAD; Ivoclar Vivadent AG, Schaan, Liechtenstein) with low translucency and A2 color was used. The milled abutment and printed adjacent teeth were post-processed according to the manufacturer’s instructions. A diamond bur (Cylindrical medium diamond; KG Sorensen, São Paulo, Brazil) was used to prepare the surface polish of the abutment. Five STL files were acquired for the abutment of the reference model by using a contact scanner (DS10; Renishaw plc, Gloucestershire, UK). These files were aligned and merged by using 3D reverse engineering software (Geomagic Design X; 3D Systems, Rock Hill, USA) to obtain 1 high-resolution CRM. The gingiva of the adjacent teeth and abutment was fabricated by using the tissues from the neck region of a pig to reproduce the oral environment similar to the actual clinical situation (Fig. 1A). Because edible pig tissues were used, approval of the Institutional Review Board was not required. The location of the subgingival finish line should not exceed the depth of 1-mm to confirm the trueness of IOSs according to the subgingival finish line depth^22,23,29,30^. Therefore, the finish line of the abutment was arranged according to 5 depths (0-mm, 0.25-mm, 0.5-mm, 0.75-mm, and 1-mm) (Fig. 1B). The subgingival finish line depth was confirmed by using 3D mesh viewer software (3Shape 3D viewer, 3Shape, Copenhagen, Denmark) and a periodontal probe (CP 15 UNC; HU-Friedy, CHI, USA). For gingival retraction, gingival displacement cords (Z-Twist Weave Retraction Cords; GINGI-PAK, Camarillo, CA, USA) were inserted into the gingival sulcus under the margin of the abutment (Fig. 1C). The gingival retraction was manipulated by using a cord packer (Packing1; atria, Seoul, Republic of Korea). To calculate the mean and standard deviation (SD) of the subgingival finish line depth at random locations, vernier calipers (500–151-30; Mitutoyo, Takatsu-ku, Japan) were used. The subgingival finish line depth were 0.2521 ± 0.0039 mm at 0.25-mm, 0.5008 ± 0.0005 mm at 0.5-mm, 0.7507 ± 0.0008 mm at 0.75-mm, and 1.0006 ± 0.0005 mm at 1-mm.

CTMs were obtained by scanning the reference model with two types of IOSs: i500 (MEDIT, Seoul, Republic of Korea) and CS3600 (Carestream Dental, Atlanta, USA) (Fig. 1D). To confirm the effect of the subgingival finish line depth, IOSs were used to scan at 0-mm, 0.25-mm, 0.5-mm, 0.75-mm, and 1-mm. After scanning with 2 IOSs without gingival retraction, the gingival retraction was performed immediately and scanned. 400 CTMs were obtained by scanning 20 times before gingival retraction and 20 times after gingival retraction for each IOS depending on the depth. To prevent the gingiva replaced with pig tissue from drying and shrinking, distilled water was wetted in the gingiva region before scanning, and moisture on the abutment was removed so as not to affect the scanning environment. All areas except the area to be evaluated were removed by using CAD software (Meshmixer; Autodesk, San Rafael, USA) so that the acquired CTM was not disturbed during the superimposition of data. All scanning was performed at an ambient temperature of 23 ± 2 °C and a humidity of 50 ± 5% in consideration of the oral environment. All scanning procedures were evaluated by a trained investigator.

Scan data of CRM and CTM were 3D analyzed by using 3D inspection software (Geomagic Control X; 3D Systems, Rock Hill, USA). For detailed analysis, the occlusal region, axial region, and marginal region were evaluated separately. 3D analysis was performed on the prepared teeth (all regions above the finish line of abutment), occlusal region (the region down to 1 mm below the occlusal plane), axial region (between the occlusal region and the marginal region), and marginal region (the region up to 1 mm above the finish line) of the abutment. CRM and CTM were aligned by using best-fit alignment, and the correspondence between CRM and CTM was evaluated by sing the 3D comparison function. The root mean square (RMS) was calculated based on all cloud points of CRM by using the following formula:

$$RMS=\frac{1}{\sqrt{n}}\cdot \sqrt{{\sum }_{i=1}^{n}{\left({X}_{1,i}-{X}_{2,i}\right)}^{2},}$$

where ${X}_{1,i}$ indicates a measurement point at $i$th in CRM and ${X}_{2,i}$ indicates a measurement point at $i$th in CTM. n is the number of all points evaluated. Therefore, the RMS value is the absolute average distance of all cloud points and means the degree of agreement between CRM and CTM.

CAD software (Meshmixer) was used to evaluate the difference in surface area of the scanned surface area according to the subgingival finish line depth. After loading the saved CRM and CTM data into CAD software (Meshmixer), all areas except for the area of the prepared teeth existing in the upper part based on the finish line were removed. The surface area of CRM and CTM was measured by using the stability function. The difference in surface area was evaluated by calculating the difference between the measured CRM surface area and CTM surface area data. All measured surface area data were recorded, analyzed, and evaluated.

All data were analyzed by using SPSS statistical software (release 23.0; IBM Corp, Chicago, IL, USA) (α = 0.05). One-way analysis of variance (ANOVA) and the Tukey HSD test were used to compare the trueness of IOSs according to the subgingival finish line depth and according to the evaluated region. The paired *t* test was used to compare the trueness of IOSs with and without gingival retraction, and an independent *t* test was used to compare the trueness according to the IOSs. Two- and three-way ANOVAs were used to verify the interaction effects.

## Results

**Figure 2:**
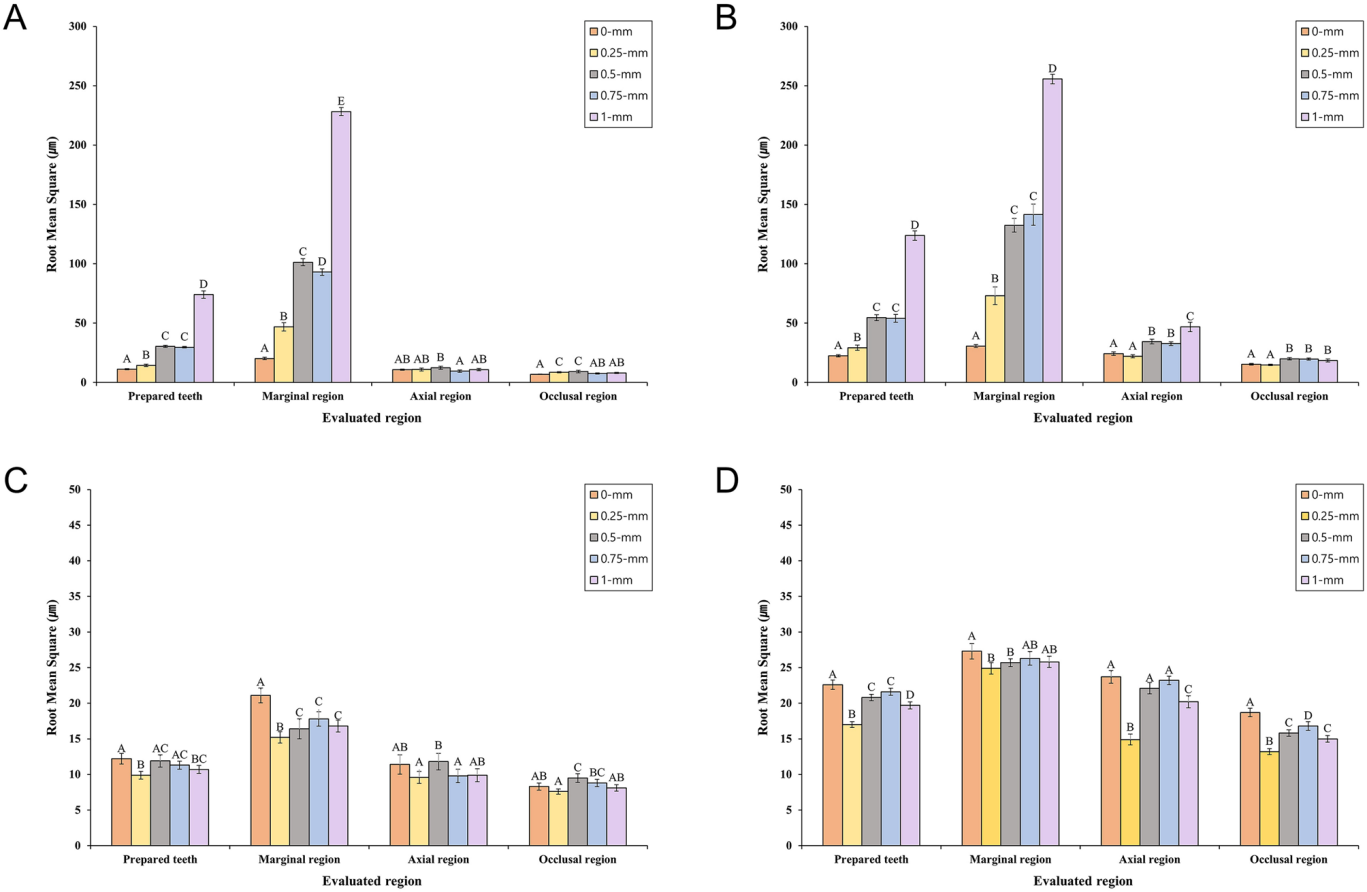
Comparison of RMS values according to subgingival finish line depth. (**A**) CS3600 IOS and (**B**) i500 IOS are without gingival displacement cords. (**C**) CS3600 IOS and (**D**) i500 IOS are with gingival displacement cords. Identical letters indicate that difference between groups is not significant (*P* ≥ 0.05).

Table 1 and Fig. 2A,B present a comparison of the trueness of IOSs at the subgingival finish line location without gingival retraction. When the gingival cord was not used, we obtained the following results: both IOSs had significant differences in trueness according to the evaluated region (prepared teeth, occlusal, axial, and marginal) of the abutment (*P* < 0.001); both IOSs had significant differences in trueness according to the depth of the subgingival finish line in the prepared teeth (*P* < 0.001), But there was no significant difference between 0.5-mm and 0.75-mm; both IOSs had a significant difference in trueness according to the subgingival finish line depth in the marginal region (*P* < 0.001); the trueness of both IOSs exceeded 100 μm when deeper than 0.5-mm in the marginal region; both IOSs showed the worst trueness at a subgingival finish line depth of 1-mm (CS3600: 228.2 ± 6.7 µm, i500: 255.6 ± 8.0 µm). When comparing the trueness of CS3600 and i500, a significant difference was observed between the 2 IOSs (*P* < 0.001). CS3600 showed significantly higher trueness than i500 (*P* < 0.001).

**Table 1:** Comparison of trueness according to subgingival finish line depth without gingival displacement cord.

IOS	Evaluated region	Depth (mm)	Mean	SD	95% CI		Minimum	Maximum	*P*^†^	*P*^††^	*P*^†††^
					Lower	Upper					
CS3600	Prepared teeth	0	11.2^A^	0.9	10.7	11.6	10.1	12.9	< 0.001*	< 0.001*	< 0.001**
		0.25	14.4^B^	1.9	13.5	15.3	11.7	17.7			
		0.5	30.4^C^	1.9	29.5	31.3	25.4	35.3			
		0.75	29.7^C^	1.3	29.1	30.4	28.0	31.9			
		1	74.0^D^	6.1	71.1	76.9	63.8	90.6			
	Marginal region	0	20.2^A^	2.1	19.1	21.2	17.4	24.1	< 0.001*		
		0.25	46.9^B^	6.8	43.6	50.1	38.4	60.5			
		0.5	101.3^C^	6.1	98.4	104.2	89.0	115.3			
		0.75	93.0^D^	5.7	90.3	95.7	84.7	103.8			
		1	228.2^E^	6.7	225.1	231.4	219.5	239.2			
	Axial region	0	10.8^AB^	1.1	10.2	11.3	8.4	12.5	0.002*		
		0.25	10.9^AB^	2.3	9.8	12.0	6.9	15.2			
		0.5	12.4^B^	2.6	11.2	13.7	9.6	21.4			
		0.75	9.5^A^	2.1	8.5	10.5	7.2	16.7			
		1	10.8^AB^	2.0	9.8	11.7	7.7	14.4			
	Occlusal region	0	6.9^A^	0.5	6.6	7.1	5.9	8.0	< 0.001*		
		0.25	8.6^BC^	1.0	8.1	9.1	7.3	11.4			
		0.5	9.2^C^	2.1	8.2	10.2	7.6	17.7			
		0.75	7.7^AB^	1.0	7.2	8.2	6.1	10.5			
		1	8.0^AB^	1.0	7.5	8.5	6.6	11.1			
i500	Prepared teeth	0	22.5^A^	1.9	21.6	23.3	20.3	27.8	< 0.001*	< 0.001*	
		0.25	29.3^B^	4.5	27.2	31.4	23.1	41.2			
		0.5	54.6^C^	5.1	52.2	57.0	46.6	64.3			
		0.75	54.0^C^	6.7	50.8	57.1	40.9	65.6			
		1	123.8^D^	7.9	120.1	127.5	110.0	146.2			
	Marginal region	0	30.7^A^	2.4	29.5	31.8	27.3	36.6	< 0.001*		
		0.25	73.0^B^	14.9	66.0	80.0	49.7	105.1			
		0.5	132.4^C^	11.5	127.0	137.9	108.2	152.1			
		0.75	141.4^C^	17.8	133.1	149.8	109.5	170.8			
		1	255.6^D^	8.0	251.9	259.4	242.5	271.2			
	Axial region	0	24.2^A^	3.0	22.8	25.7	20.9	32.5	< 0.001*		
		0.25	22.0^A^	2.6	20.7	23.2	17.6	28.9			
		0.5	34.4^B^	4.0	32.5	36.3	28.0	41.2			
		0.75	32.7^B^	3.2	31.2	34.2	26.0	37.9			
		1	46.8^C^	7.9	43.1	50.5	32.3	62.6			
	Occlusal region	0	15.4^A^	1.4	14.7	16.1	12.9	18.5	< 0.001*		
		0.25	14.8^A^	1.2	14.2	15.4	11.8	17.0			
		0.5	20.0^B^	2.0	19.1	21.0	16.7	23.5			
		0.75	19.7^B^	1.7	18.9	20.5	16.9	23.3			
		1	18.5^B^	2.7	17.2	19.8	14.6	23.5			

Significant difference by *one-way ANOVA and **independent *t* test (*P* < 0.05). Same superscript uppercase letters are not significantly different according to Tukey HSD test (*P* < 0.05). ^†^Comparison of trueness according to depth, ^††^comparison of trueness according to evaluated region, and ^†††^comparison of trueness according to intraoral scanner.

Table 2 and Fig. 2C,D present a comparison of the trueness of IOSs at the subgingival finish line location with gingival retraction. When the gingival cord was used, we obtained the following results: it showed significantly higher trueness than when gingival cord was not used (*P* < 0.001); the trueness of both IOSs did not exceed 100 μm regardless of the subgingival finish line depth and evaluated region. When comparing the trueness of CS3600 and i500, a significant difference was observed between the 2 IOSs (*P* < 0.001). CS3600 showed significantly higher trueness than i500 (*P* < 0.001).

**Table 2:** Comparison of trueness according to depth of subgingival finish line with gingival displacement cord.

IOS	Evaluated region	Depth (mm)	Mean	SD	95% CI		Minimum	Maximum	*P*^†^	*P*^††^	*P*^†††^
					Lower	Upper					
CS3600	Prepared teeth	0	12.2^A^	1.5	11.4	12.9	10.2	17.9	< 0.001*	< 0.001*	< 0.001**
		0.25	9.9^B^	1.1	9.3	10.4	8.0	12.3			
		0.5	11.9^AC^	1.7	11.1	12.7	9.0	15.8			
		0.75	11.3^AC^	1.1	10.8	11.9	9.2	13.7			
		1	10.7^BC^	1.1	10.2	11.3	8.8	13.2			
	Marginal region	0	21.1^A^	2.1	20.1	22.1	17.6	26.3	< 0.001*		
		0.25	15.2^B^	1.6	14.4	15.9	13.0	19.1			
		0.5	16.4^BC^	2.8	15.1	17.8	11.9	22.5			
		0.75	17.8^C^	2.0	16.9	18.8	14.8	22.7			
		1	16.8^BC^	1.7	16.0	17.6	14.3	20.4			
	Axial region	0	11.4^AB^	2.7	10.1	12.7	8.9	22.3	0.003*		
		0.25	9.6^A^	1.7	8.8	10.4	6.9	13.4			
		0.5	11.8^B^	2.3	10.7	12.9	8.1	16.9			
		0.75	9.8^A^	1.9	8.9	10.7	6.3	14.1			
		1	9.9^AB^	1.8	9.1	10.8	6.8	13.7			
	Occlusal region	0	8.3^AB^	1.0	7.8	8.8	6.9	11.9	< 0.001*		
		0.25	7.6^A^	0.7	7.3	7.9	6.7	9.4			
		0.5	9.5^C^	1.2	8.9	10.1	7.8	12.0			
		0.75	8.8^BC^	1.0	8.3	9.3	6.6	10.5			
		1	8.1^AB^	0.9	7.6	8.5	6.7	10.1			
i500	Prepared teeth	0	22.6^A^	1.3	22.0	23.3	20.0	25.1	< 0.001*	< 0.001*	
		0.25	17.0^B^	0.8	16.6	17.4	15.7	18.5			
		0.5	20.8^C^	0.9	20.4	21.2	18.7	23.0			
		0.75	21.6^C^	1.0	21.1	22.1	19.5	24.5			
		1	19.7^D^	1.0	19.2	20.2	17.2	21.4			
	Marginal region	0	27.3^A^	2.2	26.2	28.3	23.0	30.4	0.001*		
		0.25	24.9^B^	1.6	24.1	25.7	22.7	28.2			
		0.5	25.7^B^	1.1	25.2	26.2	23.6	27.2			
		0.75	26.3^AB^	1.9	25.4	27.2	23.3	29.3			
		1	25.8^AB^	1.6	25.0	26.5	23.3	29.1			
	Axial region	0	23.7^A^	1.8	22.9	24.6	20.2	27.2	< 0.001*		
		0.25	14.9^B^	1.5	14.2	15.6	12.7	19.3			
		0.5	22.1^A^	1.6	21.4	22.9	18.3	25.6			
		0.75	23.2^A^	1.2	22.6	23.8	21.7	27.2			
		1	20.2^C^	1.7	19.4	21.0	16.4	22.1			
	Occlusal region	0	18.7^A^	1.2	18.1	19.3	16.7	20.5	< 0.001*		
		0.25	13.2^B^	0.8	12.8	13.5	11.4	15.0			
		0.5	15.8^C^	0.9	15.4	16.3	13.7	17.7			
		0.75	16.8^D^	1.2	16.2	17.4	14.4	19.2			
		1	15.0^C^	0.9	14.6	15.5	13.4	16.9			

Significant difference by *one-way ANOVA and **independent *t* test (*P* < 0.05). Same superscript uppercase letters are not significantly different according to Tukey HSD test (*P* < 0.05). ^†^Comparison of trueness according to depth, ^††^comparison of trueness according to evaluated region, and ^†††^comparison of trueness according to intraoral scanner.

Table 3 shows the interaction effects on trueness according to the type of IOS, the subgingival finish line depth, the use of the gingival cord, and the evaluation region. When all the evaluated variables were compared, significant differences were observed in RMS values (*P* < 0.001). Significant differences was found when the interaction effect for each of the 3 sources (Depth of subgingival finish line, gingival displacement cord, and evaluated region) was observed for the intraoral scanner (*P* < 0.001). Significant differences was found when the interaction effect for each of the 2 sources (depth of subgingival finish line, evaluated region) was observed ofr the gingival displacement cord (*P* < 0.001). Significant difference was observed when the interaction effects on the intraoral scanner, depth of subgingival finish line, and gingival displacement cord were observed (*P* < 0.001).

**Table 3:** Results of ANOVA of intraoral scanner, depth of subgingival finish line, gingival displacement cord, and evaluated region.

Source	*P* (order of RMS values)
Intraoral scanner	< 0.001* (CS3600 < i500)
Depth of subgingival finish line	< 0.001** (0-mm < 0.25-mm < 0.75-mm = 0.5-mm < 1-mm)
Gingival displacement cord	< 0.001*** (with cord < without cord)
Evaluated region	< 0.001** (occlusal < axial < prepared teeth < marginal)
Intraoral scanner × depth of subgingival finish line	< 0.001****
Intraoral scanner × gingival displacement cord	< 0.001****
Intraoral scanner × evaluated region	< 0.001****
Gingival displacement cord × depth of subgingival finish line	< 0.001****
Gingival displacement cord × evaluated region	< 0.001****
Intraoral scanner × depth of subgingival finish line × gingival displacement cord	< 0.001*****

Significant difference by *independent *t* test, **one-way ANOVA, ***paired *t* test, ****two-way ANOVA, and *****three-way ANOVA (*P* < 0.05).

Table 4 presents a comparison of the difference in surface area between CRM and CTM according to the subgingival finish line depth. When the gingival cord was not used, both IOSs had a significant difference in the surface area of the abutment according to the subgingival finish line depth (*P* < 0.001). The difference in surface area was the highest at the subgingival finish line depth of 1-mm (CS3600: 15.4 mm^2^, i500: 16.6 mm^2^). When using the gingival cord, it was confirmed that the reduction in the surface area of the abutment was reduced for both IOSs (Table 4). However, even with the use of the gingival cord, it was confirmed that the surface area of the scanned data decreased when comparing CRM and CTM.

**Table 4:** Comparison of surface area difference according to depth of subgingival finish line.

IOS	With or without cord	Depth (mm)	Mean	SD	95% CI		Minimum	Maximum	*P*^†^	*P*^††^	*P*^†††^
					Lower	Upper					
CS3600	Without cord	0	7.7^A^	0.9	7.3	8.1	5.8	9.1	< 0.001*	< 0.001**	< 0.001***
		0.25	7.1^A^	0.6	6.8	7.4	5.8	8.3			
		0.5	10.8^B^	1.2	10.2	11.4	8.6	13.4			
		0.75	9.5^C^	1.1	9.0	10.1	7.7	11.9			
		1	15.4^D^	0.6	15.0	15.7	13.9	17.0			
	With cord	0	5.5^A^	0.6	5.2	5.8	4.3	7.0	< 0.001*		
		0.25	3.4^A^	0.3	3.3	3.6	2.7	4.1			
		0.5	3.7^B^	0.5	3.4	3.9	2.9	4.7			
		0.75	3.6^C^	0.7	3.2	3.9	2.2	5.1			
		1	2.2^D^	0.3	2.0	2.3	1.7	2.9			
i500	Without cord	0	4.8^A^	2.1	3.8	5.8	1.0	7.8	< 0.001*	< 0.001**	
		0.25	8.4^B^	0.9	8.0	8.9	6.4	10.4			
		0.5	14.3^C^	0.9	13.9	14.8	12.5	15.9			
		0.75	13.0^D^	1.3	12.3	13.6	10.5	15.8			
		1	16.6^E^	0.9	16.2	17.1	14.4	18.5			
	With cord	0	6.8^A^	1.2	6.2	7.4	4.8	8.9	0.002*		
		0.25	7.2^AB^	0.8	6.8	7.6	5.9	8.5			
		0.5	7.4^AB^	0.8	7.0	7.8	6.1	9.3			
		0.75	7.8^B^	0.5	7.5	8.0	7.0	8.8			
		1	7.7^B^	0.6	7.3	8.0	6.2	8.7			

Significant difference by *one-way ANOVA, ** paired *t* test, and *** independent *t* test (*P* < 0.05). ^†^Comparison of trueness according to depth, ^††^comparison of trueness according to gingival displacement cord usage, and ^†††^comparison of trueness according to intraoral scanner. Same superscript uppercase letters are not significantly different according to Tukey HSD test (*P* < 0.05).

## Discussion

In this study, there was a significant difference in the trueness of the IOSs according to the depth (0-mm, 0.25-mm, 0.5-mm, 0.75-mm, and 1-mm) of the subgingival finish line. Previous studies have confirmed the results of the accuracy of IOSs, but studies on the trueness evaluation of IOSs according to the location of the finish line are still insufficient. Brawek et al.^33^ and Shim et al.^34^ reported that the clinically recommended scan accuracy is less than 100 µm. Son et al.^19^ reported that the trueness of the marginal region at the location of the subgingival finish line (0.5-mm below the level of the gingival) was the worst. In this study, with positioning the subgingival finish line at 0.5-mm depth, the trueness of both IOSs exceeded 100 µm in the marginal region (CS3600: 101.3 ± 6.1 µm; i500: 132.4 ± 11.5 µm). Also, it showed the worst trueness at 1-mm depth of the marginal region (CS3600: 228.2 ± 6.7 µm; i500: 255.6 ± 8.0 µm). Therefore, the first null hypothesis was rejected.

In this study, there was a significant difference in the trueness of IOSs according to with and without gingival retraction. Therefore, the second null hypothesis was rejected. Son et al.^19^ reported that the trueness of both IOSs were improved when using a gingival displacement cord. Similarly in this study, the trueness of both IOSs did not exceed 100 µm in all areas regardless of the subgingival finish line depth when using gingival displacement cords. In this study, the trueness was improved by 90% (CS3600: 92%, i500: 89%) when using gingival displacement cords. Therefore, for the subgingival finish line, the use of gingival displacement cords is recommended for IOS.

Many previous studies have performed in vitro studies on the accuracy of IOSs^19,21,35–37^. Son et al.^19^ conducted an experiment by adding a small amount of red pigment to semitransparent silicone to form the gingiva of adjacent teeth and abutment for gingival reappearance. Marotti et al.^37^ collected gingival tissue from the buccal region of pig mandibles to reproduce the oral environment and formed the gingiva. In this study, the gingiva of the adjacent teeth and abutment was formed by using the tissues of the neck region of a pig for similarity with the clinical environment. However, there are limitations because factors such as lips and cheek interference and tongue that appear in the actual oral environment are not considered.

**Figure 3:**
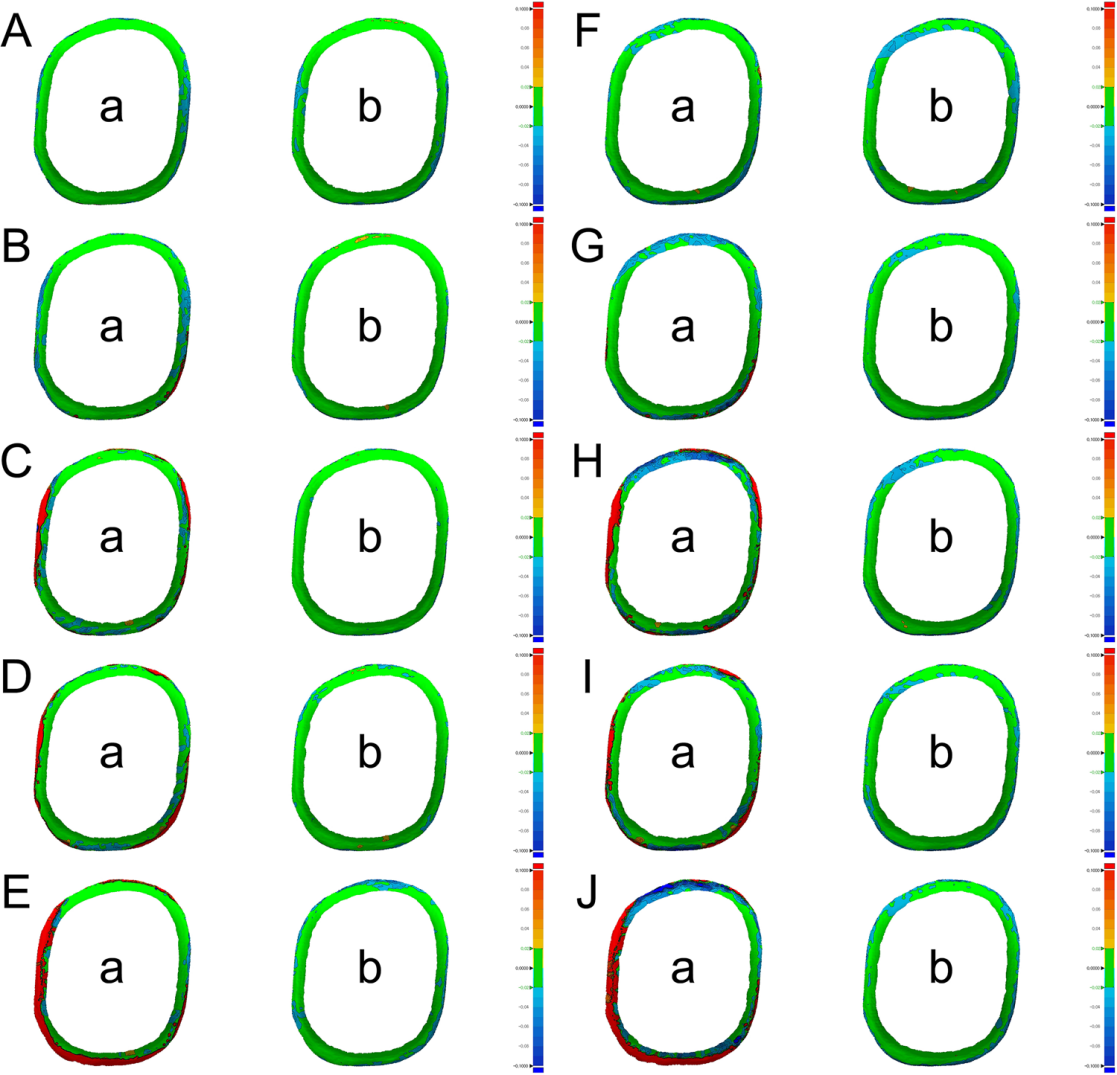
Comparison of color difference map of CS3600 and i500 IOSs according to subgingival finish line depth. (**A**) Depth of 0-mm with CS3600. (**B**) Depth of 0.25-mm with CS3600. (**C**) Depth of 0.5-mm with CS3600. (**D**) Depth of 0.75-mm with CS3600. (**E**) Depth of 1-mm with CS3600. (**F**) Depth of 0-mm with i500. (**G**) Depth of 0.25-mm with i500. (**H**) Depth of 0.5-mm with i500. (I) Depth of 0.75-mm with i500. (**J**) Depth of 1-mm with i500. (**a**) Without gingival retraction. (**b**) With gingival retraction.

**Figure 4:**
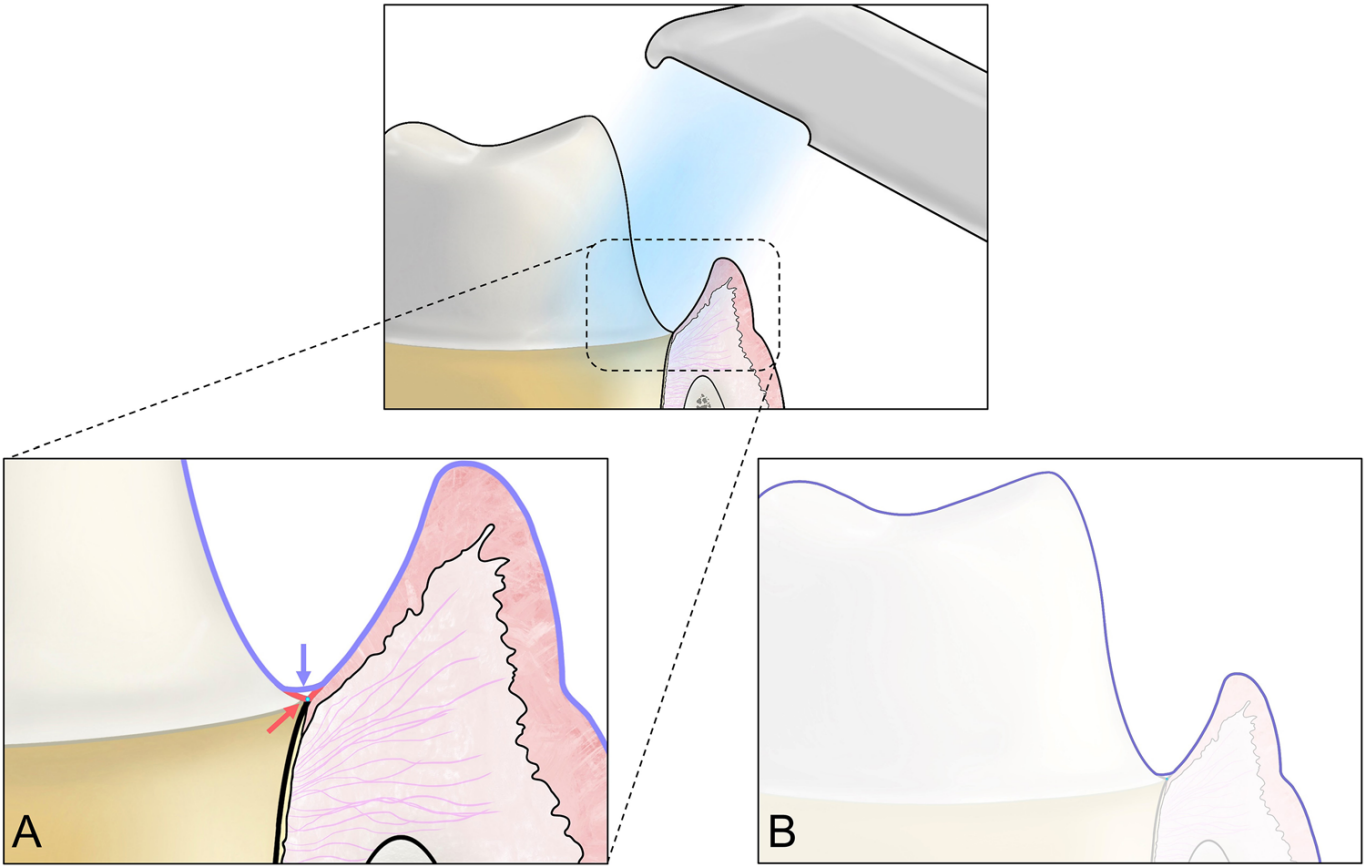
Procedure for obtaining inaccurate finish line data in process of scanning subgingival finish line using IOS. (**A**) Red arrow and line indicate actual finish line and surface and purple arrow and line indicate scanned finish line and surface. (**B**) Surface data scanned using IOS.

Nedelcu et al.^14^ reported the accuracy of IOSs in the supragingival finish line and subgingival finish line of abutments. The accuracy was visualized and analyzed through the color difference map. It was found that the positive deviation was higher in the marginal region when acquiring scan data of subgingival finish line with some IOSs. It has been reported that this positive deviation of the finish line may cause short margins and inaccurate fit during prosthesis fabrication. In this study Fig. 3 shows the 3D difference comparing the marginal regions of CRM and CTM as a color difference map. Both IOSs showed similar differences. When gingival displacement cords were not used, it was observed that the red zones in the marginal region widened as the depth increased (Fig. 3). These results may be due to the characteristics of the optical scanner to record only the visible region and the data processing technology to smoothly compensate the sharp or low point clouds acquisition region (Fig. 4)^38,39^. Additionally, this study showed that when the gingival displacement cord was inserted, almost no positive deviation was observed at all depths of the subgingival finish line and a small amount of negative deviation was observed in the edge region of the finish line (Fig. 3). In previous studies, scanners were more accurate when scanning soft areas than when scanning sharp areas.^38^ This is because the software for postprocessing of the scanner’s scan data is changed to edges shorter than the original length by connecting and smoothly correcting gaps between the outermost points when scanning the sharp edges of the finish line^38^. Therefore, the study results show that the surface area of the abutment is reduced because it was corrected by the scanner software when scanning the finish line even with the gingival cord. However, even though there was a decrease in the surface area of abutment when using the gingival cord, the trueness of the marginal region did not exceed 100 µm, so the trueness of IOSs was within the clinically acceptable range. Thus, further studies for better trueness are needed since the trueness was negatively affected by the characteristics of the IOS.

Although this in vitro study reproduced the clinical environment, the actual oral cavity could not be completely reproduced because the factors that could interfere with the scan were removed. Additional clinical trials should be performed considering factors such as the effects of chemical and physical gingival retraction and the presence or absence of saliva. Also, additional research is needed to confirm the fit of the prosthesis fabricated through the scan data since only the trueness of the IOSs was evaluated.

## Conclusion

Within the limitations of this in vitro study, the following conclusions can be drawn:

1. When the gingival displacement cord was not used, the trueness of IOSs decreased as the subgingival finish line depth increased.
2. The IOSs (CS3600 and i500) used in this study showed clinically acceptable scan trueness (< 100 µm) at a depth of up to 0.25-mm of subgingival finish line without gingival displacement cord but showed clinically acceptable scan trueness at a depth of up to 1-mm when the gingival displacement cord was used.
3. With the increase of the subgingival finish line depth without gingival displacement cord, the surface area of the abutment decreased.
4. The use of the gingival displacement cord improved surface area reduction, but the IOSs (CS3600 and i500) used in this study decreased all the surface areas of the abutments in the subgingival finish line regardless of the use of the gingival displacement cord.
5. The use of the gingival displacement cord improved the scanning trueness by 90%. Therefore, it is necessary to use the gingival displacement cord according to the clinical situation to improve scan trueness at the subgingival finish line.

## Data Availability

The datasets used and analyzed during the current study available from the corresponding author on reasonable request.

## References

[1] Rödiger M, Heinitz A, Bürgers R, Rinke S (2017). Fitting accuracy of zirconia single crowns produced via digital and conventional impressions-a clinical comparative study. Clin. Oral Investig..

[2] Christensen GJ (2009). Impressions are changing: Deciding on conventional, digital or digital plus in-office milling. J. Am. Dent. Assoc..

[3] Renne W (2017). Evaluation of the accuracy of 7 digital scanners: An in vitro analysis based on 3-dimensional comparisons. J. Prosthet. Dent..

[4] Park JM, Kim RJY, Lee KW (2020). Comparative reproducibility analysis of 6 intraoral scanners used on complex intracoronal preparations. J. Prosthet. Dent..

[5] Ahlholm P, Sipilä K, Vallittu P, Jakonen M, Kotiranta U (2018). Digital versus conventional impressions in fixed prosthodontics: A review. J. Prosthodont..

[6] Park GH, Son K, Lee KB (2019). Feasibility of using an intraoral scanner for a complete-arch digital scan. J. Prosthet. Dent..

[7] Chochlidakis KM (2016). Digital versus conventional impressions for fixed prosthodontics: A systematic review and meta-analysis. J. Prosthet. Dent..

[8] Hickel R, Manhart J (2001). Longevity of restorations in posterior teeth and reasons for failure. J. Adhes. Dent..

[9] Mandelli F, Ferrini F, Gastaldi G, Gherlone E, Ferrari M (2017). Improvement of a digital impression with conventional materials: Overcoming intraoral scanner limitations. Int. J. Prosthodont..

[10] Son K, Jin MU, Lee KB (2021). Feasibility of using an intraoral scanner for a complete-arch digital scan, part 2: A comparison of scan strategies. J. Prosthet. Dent..

[11] Müller P, Ender A, Joda T, Katsoulis J (2016). Impact of digital intraoral scan strategies on the impression accuracy using the TRIOS pod scanner. Quintessence Int..

[12] Revilla-León M (2020). Intraoral digital scans—part 1: Influence of ambient scanning light conditions on the accuracy (trueness and precision) of different intraoral scanners. J. Prosthet. Dent..

[13] Revilla-León M (2020). Intraoral digital scans: Part 2—influence of ambient scanning light conditions on the mesh quality of different intraoral scanners. J. Prosthet. Dent..

[14] Nedelcu R, Olsson P, Nyström I, Thor A (2018). Finish line distinctness and accuracy in 7 intraoral scanners versus conventional impression: An in vitro descriptive comparison. BMC Oral Health.

[15] Patzelt SB, Emmanouilidi A, Stampf S, Sturb JR, Att W (2014). Accuracy of full-arch scans using intraoral scanners. Clin. Oral Investig..

[16] Ender A, Mehl A (2013). Accuracy of complete-arch dental impressions: A new method of measuring trueness and precision. J. Prosthet. Dent..

[17] Chen Y (2022). Influence of liquid on the tooth surface on the accuracy of intraoral scanners: An in vitro study. J. Prosthodont..

[18] Song J, Kim M (2020). Accuracy on scanned images of full arch models with orthodontic brackets by various intraoral scanners in the presence of artificial saliva. BioMed Res. Int..

[19] Son K, Lee KB (2021). Effect of finish line locations of tooth preparation on the accuracy of intraoral scanners. Int. J. Comput. Dent..

[20] Koulivand S, Ghodsi S, Siadat H, Alikhasi M (2020). A clinical comparison of digital and conventional impression techniques regarding finish line locations and impression time. J. Esthet. Restor. Dent..

[21] Son K, Son YT, Lee JM, Lee KB (2021). Marginal and internal fit and intaglio surface trueness of interim crowns fabricated from tooth preparation of four finish line locations. Sci. Rep..

[22] Seymour K, Zou L, Samarawickrama DY, Lynch E (1996). Assessment of shoulder dimensions and angles of porcelain bonded to metal crown preparations. J. Prosthet. Dent..

[23] Scutellà F, Weinstein T, Zucchelli G, Testori T, Del Fabbro M (2017). A Retrospective periodontal assessment of 137 teeth after featheredge preparation and gingittage. Int. J. Periodontics Restor. Dent..

[24] Orkin DA, Reddy J, Bradshaw D (1987). The relationship of the position of crown margins to gingival health. J. Prosthet. Dent..

[25] Brägger U, Lauchenauer D, Lang NP (1992). Surgical crown lengthening of the clinical crown. J. Clin. Periodontol..

[26] Bowley JF, Sun AF, Barouch KK (2004). Effect of margin location on crown preparation resistance form. J. Prosthet. Dent..

[27] Waerhaug J (1953). Tissue reactions around artificial crowns. J. Periodontol..

[28] Goodacre CJ, Campagni WV, Aquilino SA (2001). Tooth preparations for complete crowns: An art form based on scientific principles. J. Prosthet. Dent..

[29] Ferencz JL (1991). Maintaining and enhancing gingival architecture in fixed prosthodontics. J. Prosthet. Dent..

[30] Padbury A, Eber R, Wang HL (2003). Interactions between the gingiva and the margin of restorations. J. Clin. Periodontol..

[31] Zoellner A, Brägger U, Fellmann V, Gaengler P (2000). Correlation between clinical scoring of secondary caries at crown margins and histologically assessed extent of the lesions. Int. J. Prosthodont..

[32] Anadioti E (2014). 3D and 2D marginal fit of pressed and CAD/CAM lithium disilicate crowns made from digital and conventional impressions. J. Prosthodont..

[33] Brawek PK, Wolfart S, Endres L, Kirsten A, Reich S (2013). The clinical accuracy of single crowns exclusively fabricated by digital workflow—the comparison of two systems. Clin. Oral. Investig..

[34] Shim JS (2015). Effect of software version and parameter settings on the marginal and internal adaptation of crowns fabricated with the CAD/CAM system. J. Appl. Oral Sci..

[35] Markarian RA, Vasconcelos E, Kim JH, Cortes ARG (2021). Influence of gingival contour on marginal fit of CAD–CAM zirconia copings on implant stock abutments. Eur. J. Prosthodont. Restor. Dent..

[36] Rapone B (2020). The accuracy of three intraoral scanners in the oral environment with and without saliva: A comparative study. Appl. Sci..

[37] Marotti J (2019). Impression of subgingival dental preparation can be taken with ultrasound. Ultrasound Med. Biol..

[38] González de Villaumbrosia P, Martínez-Rus F, García-Orejas A, Salido MP, Pradíes G (2016). In vitro comparison of the accuracy (trueness and precision) of six extraoral dental scanners with different scanning technologies. J. Prosthet. Dent..

[39] Medina-Sotomayor P, Pascual-Moscardó A, Camps I (2018). Relationship between resolution and accuracy of four intraoral scanners in complete-arch impressions. J. Clin. Exp. Dent..

